# Religious and Spiritual Factors in Depression: Review and Integration of the Research

**DOI:** 10.1155/2012/962860

**Published:** 2012-08-15

**Authors:** Raphael Bonelli, Rachel E. Dew, Harold G. Koenig, David H. Rosmarin, Sasan Vasegh

**Affiliations:** ^1^Departments of Psychiatry and Neurology, Sigmund Freud University, 1030 Vienna, Austria; ^2^Division of Child Adolescent Psychiatry, Duke University Medical Center, Durham, NC 27710, USA; ^3^Center for Spirituality, Theology and Health, Duke University Medical Center, Box 3400, Durham, NC 27705, USA; ^4^Department of Medicine, King Abdulaziz University (KAU), Jeddah 21589, Saudi Arabia; ^5^Department of Psychiatry, Harvard Medical School, Boston, MA 02215, USA; ^6^Department of Psychiatry, Ilam University of Medical Sciences, Tehran, Iran

## Abstract

Depressive symptoms and religious/spiritual (R/S) practices are widespread around the world, but their intersection has received relatively little attention from mainstream mental health professionals. This paper reviews and synthesizes quantitative research examining relationships between R/S involvement and depressive symptoms or disorders during the last 50 years (1962 to 2011). At least 444 studies have now quantitatively examined these relationships. Of those, over 60% report less depression and faster remission from depression in those more R/S or a reduction in depression severity in response to an R/S intervention. In contrast, only 6% report greater depression. Of the 178 most methodologically rigorous studies, 119 (67%) find inverse relationships between R/S and depression. Religious beliefs and practices may help people to cope better with stressful life circumstances, give meaning and hope, and surround depressed persons with a supportive community. In some populations or individuals, however, religious beliefs may increase guilt and lead to discouragement as people fail to live up to the high standards of their religious tradition. Understanding the role that R/S factors play in preventing depression, facilitating its resolution, or leading to greater depression will help clinicians determine whether this is a resource or a liability for individual patients.

## 1. Introduction

Depression is widespread around the world. The 12-month prevalence of major depressive disorder is 6.7% in the United States and is 2.0% for severe depression [1]. Depressive disorder has an enormous impact on a person's ability to function at work, in relationships, and in other areas of life. The World Health Organization projects that, by the year 2020, major depression will be the world's second most debilitating condition; only cardiovascular disease will cause more disability [2]. Among persons aged 15 to 44 in the USA, major depression is now the leading cause of disability days [3]. Depression affects not only ability to function and quality of life but also physical health by driving persons to suicide (over 1 million lost lives/year worldwide [4]) or by altering vital physiological processes necessary for survival (immune, endocrine, and cardiovascular functions). In the USA alone it is estimated that depression costs over $65 billion per year [5].

Religious involvement is also common today, with surveys showing that a significant proportion of the world's population has religious beliefs and practices that are important to daily life. For example, the World Gallup Poll surveyed representative populations of 143 countries (*n* = 140,000), finding that 92 percent of people in 32 developing countries indicated religion was an important part of daily life [6]. Likewise, a survey of developed countries by Angus Reid Strategies involving 5,800 adults in Australia, Britain, Canada, China, Egypt, France, Germany, India, Israel, Italy, Japan, Lebanon, Mexico, Russia, Saudi Arabia, South Africa, Republic of Korea, Spain, Turkey, and the United States found that 48% of respondents said religion is a “very important” component of their daily lives [7]. Based on information collected on 238 countries, 13,000 ethno-linguistic peoples, 5,000 cities and 3,000 provinces by the World Christian Database, atheists make up less than 0.01% of the populations of 24 countries, less than 0.1% of the populations in 100 countries on which such data are available, and more than 5% of the population in only 9 countries (Cuba, Latvia, Uruguay, Viet Nam, China, Mongolia, Kazakhstan, Sweden, and Democratic People's Republic of Korea) [8]. With regard to the United States, the most recent Gallup Poll conducted in late 2011 found that 55% of Americans indicated that religion is very important in their lives and 26% said it is fairly important, leaving only 19% who said it was not important [9].

A large and growing volume of research suggests that religious or spiritual (R/S) beliefs and practices may be used to cope with or adapt to stressful life circumstances. Although there are many genetic, developmental, and environmental factors contributing to the onset and maintenance of depression, failure to cope with life stress is often a major underlying factor [10]. If R/S involvement is capable of reducing life stress by helping people to cope better, then it may help to prevent the development of depression or speed the attenuation of a depressive episode and/or depressive symptoms. Alternatively, R/S beliefs may also create high standards that are difficult to live up to, resulting in a sense of failure and guilt. Furthermore, those unable to live according to these standards may face rejection from their faith community, resulting in social isolation. To what extent R/S helps to buffer against depression and speed its remission or serves to bring on depression or complicate its course has been studied using research methods within the social and behavioral sciences. The purpose of this review is to summarize quantitative research on the religion-depression relationship, including randomized clinical trials that have examined the effects of religious interventions on depression. Knowing about this research will help clinicians decide whether religious beliefs of patients are a resource or a liability.

## 2. Definitions

First, however, we briefly define how we are using the terms religion and spirituality in this paper. Space here does not allow a full discussion of this complex and controversial issue, so we refer the reader to other sources [11–13]. We present here abbreviated versions of the definitions presented in the *Handbook of Religion and Health*.

  Religion involves beliefs, practices, and rituals related to the transcendent, where the transcendent is God, Allah, HaShem, or a Higher Power in Western religious traditions, or to Brahman, manifestations of Brahman, Buddha, Dao, or Ultimate Truth/Reality in Eastern traditions…Religion is a multidimensional construct that includes beliefs, behaviors, rituals, and ceremonies that may be held or practiced in private or public settings, but are in some way derived from established traditions that developed over time within a community [14]….
  Spirituality is distinguished from all other things—humanism, values, morals, and mental health—by its connection to that which is sacred, the transcendent….Spirituality includes both a search for the transcendent and the discovery of the transcendent, and so involves traveling along the path that leads from non-consideration to questioning to either staunch non-belief or belief, and if belief, then ultimately to devotion, and finally, surrender [14].

Since we are discussing research, we use the terms religion and spirituality interchangeably (R/S) for two reasons. First, there is similarity between the terms, which both involve a relationship to the transcendent. Second, whenever spirituality has been assessed using measures not contaminated by items assessing mental health (well-being, peacefulness, meaning and purpose, connectedness to others, etc.), spirituality has been assessed using questions measuring religion [15, 16].

## 3. Review of the Research

The following summary of the research findings is based on two systematic reviews conducted in 2001 and 2010 and covers a period spanning between 1962 and 2010. Every study referred to in this review is annotated and described in the appendices of two editions of the *Handbook of Religion and Health* (and for an expanded discussion of this research, which we only briefly summarize here, see the chapters focused on this topic in the *Handbook)* [17, 18].

The systematic review was conducted as follows. Computer literature searches using Medline and PsychInfo databases were conducted to systematically identify studies on the depression-religion relationship by entering the search words “religion,” “religiosity,” “religiousness,” and “spirituality,” and cross-referencing these with the search term “depression." The studies' abstracts were then examined to determine if they were qualitative or quantitative. Qualitative studies were excluded, as were those with samples sizes less than 15 unless they were experimental studies. In this manner, 444 studies were identified that quantitatively measured religious involvement or spirituality; not included here are studies of religious affiliation, which are reported separately (see below). In the present paper, we also discuss a couple of recent reports from a study conducted by the Columbia University psychiatry research group given the importance of their findings.

### 3.1. Religious Affiliation

Given its superficial nature, religious affiliation is a poor indicator of degree of religious involvement or commitment. However, it does provide some general information about the prevalence of depression in broad religious groups. In general, people of Jewish descent, Pentecostals, and those with no affiliation experience higher rates of depression than other religious groups. Higher rates of depression in people of Jewish descent, particularly those who are not actively religious, have been documented in both cross-sectional and longitudinal studies [19–21]. A variety of factors may explain why people of Jewish descent at least appear to be at higher risk. One reason may be the selective reporting of depressive symptoms. In other words, people of Jewish descent may be more likely to report depressive symptoms and seek help from mental health professionals rather than turn to maladaptive means of coping with emotional pain (e.g., people of Jewish descent also demonstrate lower rates of alcohol abuse [22]). Depression rates appear highest in Jewish people of Eastern European decent, and there has long been speculation that genetic factors may contribute to depression (melancholia agitata Hebraica) among Eastern European Jews [23]. However, Glicksman in studies examining response styles suggests that Jewish people of Eastern European descent are much more likely than Irish or Italian Catholics to express negative affect [24].

Higher rates of depression in Pentecostals may be due to people with emotional problems self-selecting themselves into Pentecostal groups because of the latter's strong focus on overcoming emotional problems (many uplifting hymns, strong emphasis on socialization, and positive content of sermons) [25]. Another reason may be the strong emphasis placed on evangelism by Pentecostals, leading to drawing of members from lower socioeconomic groups that may be at high risk for depression and other mental illnesses [26].

Higher rates of depression in those lacking a religious affiliation may be due to the absence of social support from a faith community or lack of commitment to a belief system that makes sense of traumatic events and difficult life stressors. The nonaffiliated may, however, have alternative sources of support from nonreligious communities and secular belief systems that compensate for lack of religious connections. Furthermore, even those without a formal religious affiliation may nevertheless hold quite devout religious beliefs that are expressed in nonorganizational ways.

### 3.2. Religious/Spiritual Involvement

Rather than simply focusing on affiliation, however, we are particularly interested in the relationship between level of R/S involvement (e.g., importance of belief, degree of commitment, and amount of time spent in religious activities) and depression. As noted above, at least 444 original quantitative studies examined the relationship between R/S and depression or the effects of R/S intervention on depression between 1962 and 2010. Of those, there were 414 observational studies and 30 clinical trials (Table 1). Overall, of the 444 total studies, 272 (61%) found less depression, faster recovery from depression, or a reduction in depressive symptoms in response to an R/S intervention, whereas 28 studies (6%) found the opposite.

Rather than simply present the results for all studies regardless of design or quality, the methodological rigor for each study was rated on a scale from 1 to 10 based on a scheme adapted from Cooper [27]. Cooper emphasized the definition of variables, validity, and reliability of measures, how representative the sample was, quality of the research methods, how well the execution of the study conformed to the design, appropriateness of statistical tests, and the interpretation of results. Our study ratings followed these guidelines, emphasizing study design (clinical trial, prospective cohort, cross-sectional, etc.), sampling method (random, systematic, or convenience), number of R/S measures, quality of R/S measures, quality of mental health outcome measure, contamination between outcome and R/S measures, inclusion of control variables, and quality of the statistical analyses. This method was tested for inter-rater reliability in a subgroup of 75 studies (examining relationships between R/S and depression, as well as between R/S and other mental and physical health outcomes) [17]. Direct correlation between two separate raters (Pearson's r) was 0.57. The Kappa statistic of agreement for categorizing studies as higher quality (ratings of 7 or higher) versus lower quality (ratings less than 7) was 0.49 (indicating good inter-rater agreement [28]).

Of the 444 studies, 178 (40%) were rated 7 or higher on the 1-to-10 scale. Of these methodologically more rigorous studies, 119 (67%) found less depression, faster recovery, or greater responsiveness to R/S interventions, whereas 13 studies (7%) reported the opposite. Thus, overall, 61% of studies find less depression among the more religious, and as the quality of the study increases, this proportion remains the same or increases slightly (67%). These findings are similar to those of a meta-analysis conducted by Smith and colleagues that was published in 2003 [29], which examined findings on the religion-depression relationship using data from 98,975 subjects involved in 147 studies. The average effect size (correlation) was small (*r* = −0.10) but consistent and could not be explained by gender, age, or ethnicity. Furthermore, the size of the effect was equivalent to the effect of gender on depression based on similar meta-analyses (and certainly gender is considered a major risk factor for depression). Interestingly, studies that included subjects experiencing high levels of stress found the buffering effect of religious involvement was 50% stronger (*r* = −0.15).

More recently, psychiatric epidemiologists at Columbia University have examined whether religiosity protects against depression in high risk individuals [30]. Investigators reported results from a 10-year prospective study of 114 adult offspring of depressed (*n* = 72) and nondepressed parents (*n* = 42). Religious measures at baseline were personal importance of religion or spirituality, frequency of attendance at religious services, and denomination. The outcome was the presence of major depression at the 20-year follow-up (10 years after religious measures were assessed). After controlling for the covariates gender, age, history of depression, and risk status (based on parental depression), those who indicated that religion or spirituality was highly important to them were 73% less likely to be depressed (OR = 0.27, 95% CI = 0.07–1.08, *P* = 0.06, trend). In the low-risk group without a history of depressed parents, religious variables did not predict the presence of depression at follow-up. However, in those at high risk due to parental depression, those indicating at baseline that religion or spirituality was highly important to them were 90% less likely to have major depression (OR = 0.10, 95% CI 0.01–0.92).

In a second report from this study, where the sample was expanded from 114 to 185 participants, investigators examined differences in relationships between R/S and future depression episodes based on level of exposure to negative life events (NLE) [31]. All analyses were controlled for age, gender, denomination, history of depression, and history of parental depression. In the overall sample, increased religious attendance predicted a 49% lower likelihood of mood disorder (OR = 0.51, 95% CI 0.30–0.87) and 53% lower likelihood of any psychiatric disorder (OR = 0.47, 95% CI 0.29–0.79). Attendance also reduced the effect that parental depression had on mood disorder (interaction OR = 2.13, 95% CI 1.14–3.97) and on any psychiatric disorder (interaction OR = 1.81, 95% CI 0.96–3.41, *P* < 0.07, trend). Most important, however, was the interaction found with NLEs. For high risk participants (those with depressed parents) with high exposure to NLEs, religious attendance reduced the likelihood of major depression on follow-up by 76% (OR = 0.24, 95% CI 0.06–0.94), any mood disorder by 69% (OR = 0.31, 95% CI 0.09–1.00), and any psychiatric disorder by 64% (OR = 0.36, 95% CI 0.11–1.17, *P* < 0.10, trend). Importance of religion/spirituality also reduced the odds of mood disorder in this group by 74% (OR = 0.26, 95% CI 0.07–0.94).

These latter reports underscore the role that R/S involvement may play in protecting those at high-risk for depression because of a family history of depression, the presence of negative life events, or both.

## 4. Suicide

The findings from research on R/S and depression are also consistent with research on the relationship between R/S and suicide. Depression is a well-established risk factor for suicide. Indeed, depression—along with anger, need for control, and impulsiveness—is psychological state often associated with suicide attempts and completed suicide [32]. Substance abuse is another factor, along with life stressors. One study of suicide in Finland (which has some of the highest suicide rates in the world) found that recent life events were documented in 80% of suicides [33]. If R/S involvement is related to less depression, less anger and hostility, lower rates of substance abuse, greater social support, and better coping with stress, it should not be surprising that R/S is also related to less suicide. A systematic review of this literature, presented in the 2001 and 2012 editions of *Handbook,* identified 141 studies that examined the relationship between R/S and completed suicide, attempted suicide, or attitudes toward suicide. Of those, 106 (75%) found inverse relationships (39 of the 49 highest-quality studies or 80%) [34]. Only 4 of 141 (<3%) studies found more suicide attempts, completed suicide, or positive attitudes toward suicide among people with more R/S involvement. Thus, research findings for both depression and suicide reinforce the notion that R/S involvement may serve as an important resource for some individuals at risk for depression and its most feared consequence, suicide.

## 5. Reasons for Less Depression and Suicide

The majority of studies (61%) find less depression or faster recovery from depression for those who are more R/S or a better response to an R/S intervention compared to other treatments or controls. Even a higher percentage of studies (75%) find inverse relationships between R/S and suicide attitudes, attempts, and completed suicide, and <3% find the opposite. Why is this so? We already discussed the possibility that R/S involvement may help persons to cope better. This has been reported in hundreds of both quantitative and qualitative studies where individuals enduring stressful life circumstances are asked what enables them to cope with the stress [35, 36]. For example, in one study of 330 consecutively hospitalized patients to the general medicine, cardiology and neurology services of Duke Hospital, when asked an open-ended question about what enabled them to cope with the stress of their illness, 42% spontaneously reported that it was some aspect of religious faith or activity [37]. Furthermore, R/S has been shown to predict a faster speed of remission of depression in at least three studies of hospitalized patients experiencing the stress of medical illness [38–40].

Besides helping people to cope better with life stressors, R/S involvement may reduce the likelihood that stressors will happen in the first place. Daily decisions that involve choices on how to treat others (generosity, altruism, gratefulness, and forgiveness), lifestyle practices (marital fidelity, delinquency or crime, and school performance), and health behaviors (use of alcohol, use of drugs, and disease prevention activities) may influence the psychosocial or physical stressors that a person has to deal with. Since R/S involvement has been associated with greater altruism, gratefulness, forgiveness, marital satisfaction, less delinquency/crime, better school performance, less substance abuse, and more disease prevention activities [14], it would make sense that this should result in fewer life stressors. Further, social support has been known to buffer against depression and suicide in a wide range of studies and populations since the mid-1970s [41, 42] and is likely one way that R/S helps people to cope with life stressors. A strong support system involving friends and family is a powerful resource for those facing difficult circumstances out of their control. In the *Handbook*'s systematic review of research on R/S and social support, 82 percent of quantitative studies (61 of 74) reported significant positive relationships between the two [14].

R/S involvement has also been associated with positive emotions, such as greater life satisfaction, well-being, hope, optimism, and meaning and purpose in life, feelings which help to neutralize the negative emotions that underlie depression and suicide. The *Handbook*'s systematic review found links with R/S in 256 of 326 studies (79%) on well-being/life satisfaction, 29 of 40 studies (73%) on hope, 26 of 32 studies (81%) on optimism, and 42 of 45 studies (93%) on meaning and purpose [14]. Thus, these constructs should be reflected in the associations between R/S involvement, depression, and suicide.

## 6. Reasons for More Depression

In some populations, however, it appears that R/S involvement is related to higher rates of depression. This is particularly true for religious persons who are struggling with family issues related to child problems, marital problems, abuse, or caregiving issues (R/S is more likely to be inversely related to depression in those dealing with more external problems related to finances or health issues) [43]. Failure in family life, an area of particular importance to highly religious persons because of its emphasis by religious traditions, may predispose to higher levels of guilt and greater depression.

Several high-quality studies (methodology ratings of 7 or higher on a 1-to-10 scale) published since the year 2000 have found a positive link between R/S and depression in various other settings. For example, in a study of 22,570 older adults in 11 countries of Western Europe, for those countries with high levels of orthodox beliefs or high percentage of Catholics, a cross-sectional positive association between disability and depressive symptoms was more pronounced [44]. Likewise, a 2-year prospective study of depressive symptoms in 219 couples from The Netherlands who had suffered the loss of a child, those with a religious affiliation were significantly more likely to experience depression than those without a religious affiliation [45]. Given the high value that religion places on family and children, the loss of a child may be more distressing and associated with more depression, as other research has suggested [46]. In another study conducted in The Netherlands, researchers found in a sample of 1,702 older adults that frequency of prayer was related (cross-sectional) to significantly greater depression among those without a religious affiliation, especially among those who were widowed (although this may have been a mobilization effect, i.e., people praying because they feel down) [47].

In a study conducted in Providence, Rhode Island, religious attendance and major depression were examined in a sample of 718 participants (mean age 34). Among men (*n* = 438), cross-sectional analyses revealed that those not attending religious services were 44% less likely to have major depression; in fact, compared to men who attended religious services both during youth and currently, those who changed their frequency of attendance (most of whom stopped attending) were at even lower risk of depression (OR = 0.50, 95% CI 0.31–0.83) [48]. Finally, a clinical trial examined the effects of manual-guided spiritual direction (SD) on depressive symptoms in 60 adults following inpatient detoxification for substance abuse (New Mexico). Subjects were randomly assigned to either a spiritual direction intervention or to a treatment as usual control (TAU) group that received behavioral counseling and education. At 4-month follow-up, depressive symptoms were significantly higher in the SD group compared to the TAU group (*t*[37] = −3.93, *P* < 0.001), although the difference disappeared at 8 and 12-month follow-ups.

Thus, among those with family problems, those living in Catholic countries in Europe with orthodox beliefs, couples in The Netherlands experiencing bereavement, older widowed European widows without a religious affiliation, young men from Providence Rhode Island, and psychiatric inpatients with substance abuse problems, R/S involvement appears to be associated with a greater risk of depression. Four of the six studies above were cross-sectional (preventing causal inferences), although one was prospective (but examined religious affiliation only) and one was a well-designed clinical trial. In some of the studies above, it is likely that guilt may have been aroused by R/S involvement and could help to explain some of the association. In other cases, religious involvement may have been an indicator of stress in populations characterized by low religious involvement, where turning to religion only occurs when stress levels are high and people are particularly desperate (i.e., mobilization effect).

## 7. Clinical Applications

If R/S is generally related to less depression (and suicide) and predicts a faster remission from depression over time, then could this information be of use to clinicians? Indeed, a number of randomized clinical trials have examined whether utilizing patients' R/S resources in therapy may help to speed the resolution of depression. Evidence of this sort, and the development of R/S forms of psychotherapy for depression, might increase access to therapy for many depressed persons who consider R/S important in their lives, yet fear seeking secular therapy because they are concerned that their R/S beliefs will not be respected. This has been a major barrier to R/S persons seeking professional help since time of Freud [49] and such negative attitudes have not changed much [50, 51]. A recent national survey of USA psychiatrists found that 56% never, rarely, or only sometimes inquire about religious/spiritual issues in patients with depression or anxiety, and when inquiry does occur, it often is done in the context of R/S as a cause for the psychopathology [52, 53].

Furthermore, pastoral counselors, chaplains, and even community clergy could use such therapies to help many R/S persons that they seek to help. A poorly known fact is that community clergy spend on average about 15% of their time counseling at the local level [54]. Considering that there are over 300,000 clergy in the USA alone (not including the activities of nearly 100,000 full-time nuns or chaplains), this means more than 140 million hours of therapy is provided by clergy each year—equivalent to the entire membership of the American Psychological Association providing 33 hours/week of counseling [54]. Clergy, then, are often on the front lines of mental health care but seldom receive the training to do so. Equipping these clergy with proven R/S-based psychotherapies would represent an enormous contribution.

In fact, there are several randomized clinical trials already completed that demonstrate benefit using religious or spiritually integrated psychotherapies for depression. For example, researchers examined the effectiveness of religious (Christian) cognitive-behavioral psychotherapy (RCBT) compared to conventional CBT (CCBT), ordinary pastoral counseling (PCT), and a wait-list control condition (WLC) in the treatment of depressed religious patients [55]. Fifty-nine subjects were randomized to these four groups and received 18 therapy sessions over 3 months. Only those in the RCBT condition experienced significantly lower immediate posttreatment depression scores (Beck Depression Inventory or BDI) compared to WLC. RCBT and PCT also showed trends toward lower posttreatment Hamilton Depression Rating Scale scores compared to WLC. Finally, RCT resulted in significantly better social adjustment scores (SAS) compared to the WLC (*P* < 0.001).

Furthermore, at least two randomized clinical trials have found that psychotherapy supplemented with teachings from the Koran and Islamic prayer was effective in treating depression (*n* = 64) and bereavement (*n* = 30) among religious Muslims in Malaysia, compared to traditional therapy [56, 57]. Since the year 2000, at least 22 clinical trials or experimental studies have examined the effects on depressive symptoms, including meditation, religious forgiveness therapy, mantra chanting, spiritual coping therapy, spiritual-focused therapy, spiritual history taking, a spiritual teaching program, 12-step spirituality program, spiritual direction, and a variety of other psychospiritual interventions, of which nearly two-thirds (63%) reported significant benefits [58]. A relatively recent meta-analytic review by Smith and colleagues found between-treatments effect size of 0.51 overall and 0.96 for studies which assessed for positive as well as negative outcomes [59]. Another independent review of this research by other investigators has recently confirmed the role that R/S could play in the treatment of depression and other psychiatric disorders as well [60].

Finally, there is also evidence that use of R/S therapies for depression does not have to be restricted to R/S therapists. Rather, these therapies can be delivered by secular therapists as well, sometimes even more effectively than by R/S therapists [55, 61].

## 8. Conclusions

There are certainly many factors that influence the risk of depression besides R/S, including genetic, developmental, and environmental factors. However, in the majority of studies, everything else being equal, R/S involvement is related to less depression, particularly in the context of life stress. The systematic review discussed above indicates many more studies show possible benefits from R/S compared to those that show possible harm (61% versus 6% of studies). Nevertheless, a number of high-quality studies show that R/S involvement may increase the risk of depression in certain populations (those with family problems) or may worsen the prognosis of depression (a single study in substance abusers). Interventions that utilize the R/S beliefs of patients have been tested in randomized clinical trials and shown to reduce depressive symptoms, and clinical trials are now examining the effects of religious psychotherapy against standard therapies [62]. R/S involvement appears to be related to depression in one way or another. Given the worldwide prevalence of both R/S and depression, the frequent use of R/S as a coping behavior and reported effectiveness, and the serious disability that depression causes, researchers and clinicians need to better understand how R/S impacts mental health and vice versa.

## Figures and Tables

**Table 1:** Systematic review on depression and religiosity/spirituality (1962–2010).

	Observational studies			
	Cross-sectional	Prospective	Clinical trials	
				Total (column)
Findings	*N* (%)	*N* (%)	*N* (%)	*N* (%)
Inverse (−)/improved	214 (62)	39 (56)	19 (63)	272 (61)
Positive (+)/worse	19 ( 6)	7 (10)	2 (7)	28 (6)
Mixed or no association	111 (32)	24 (34)	9 (30)	144 (32)

Total *N *	344 (100)	70 (100)	30 (100)	444 (100)
